# COVID-19 measures as an opportunity to reduce the environmental footprint in orthopaedic and trauma surgery

**DOI:** 10.3389/fsurg.2023.959639

**Published:** 2023-04-12

**Authors:** Eva-Maria Arndt, Tom Rainer Jansen, Jessica Bojko, Jonas Joachim Roos, Mari Babasiz, Thomas Martin Randau, Kristian Welle, Christof Burger, Koroush Kabir

**Affiliations:** ^1^ Department for Orthopedic and Traumatology Surgery, University Hospital Bonn, Bonn, Germany; ^2^ Department for Orthopedic and Traumatology Surgery, University Hospital Wuppertal, Wuppertal, Germany

**Keywords:** carbon footprint, telemedicine, video consultation, orthopaedic and trauma surgery, COVID-19

## Abstract

**Background:**

Climate change and its consequences on our everyday life have also tremendous impacts on public health and the health of each individual. The healthcare sector currently accounts for 4.4% of global greenhouse gas emissions. The share of the emissions in the health care system caused by the transportation sector is 7%. The study analyses the effect of video consultation on the CO_2_ emissions during the Covid-19 pandemic in an outpatient clinic of the department of orthopaedics and traumatology surgery at a German university hospital.

**Methods:**

The study participants were patients who obtained a video consultation in the period from June to December 2020 and voluntarily completed a questionnaire after the consultation. The type of transport, travel time and waiting time as well as patient satisfaction were recorded by questionnaire.

**Results:**

The study comprised 51 consultations. About 70% of respondents would have travelled to the clinic by car. The reduction in greenhouse gas emissions of video consultations compared to a face-to-face presentation was 97% in our model investigation.

**Conclusion:**

The video consultation can be a very important part of the reduction of greenhouse gas emissions in the health care system. It also saves time for the doctor and patient and can form an essential part of individual patient care.

## 1. Introduction

Climate change and its consequences on our everyday life have also tremendous impacts on public health and the health of each individual. In addition to the direct consequences of air pollution, such as respiratory diseases, the effects on health also include the indirect consequences of climate change, such as the spread of infectious diseases due to globalization, food shortages and consequently malnutrition and migration (1–3). For the year 2018, the European Environment Agency describes in its 2019 report that the premature death of 400,000 people in the EU is due to air pollution. This is explained by the increase in heart attacks (12%), lung cancer (11%) and strokes (11%) linked to air pollution. This concerns especially vulnerable people with pre-existing health conditions. By reducing greenhouse gas emissions, premature deaths also decrease (4–8).

The major part of greenhouse gases is produced by the combustion of fossil fuels. If their proportion in the atmosphere increases, this leads to an intensification of the greenhouse effect and thus to global warming. Greenhouse gases include carbon dioxide (CO_2_), methane (CH_4_), nitrous oxide (N_2_O_2_), chlorofluorocarbon (CFC) and water vapour (7). A CO_2_ equivalent (CO_2_e) is a measure of the global warming potential of a substance or the climate-damaging effect of an activity (1, 6). The healthcare sector currently accounts for 4.4% of global greenhouse gas emissions. According to the organisation “Health Care Without Harm” (HCWH), the EU's amount of global healthcare sector emissions is 12% (8). In Germany, the share of greenhouse gas emission in the health care sector is 5.2% of the total emission (5, 6). The share of greenhouse gas emissions in the health care system caused by the transportation sector is 7%, according to the data in the European Union (5, 6). In HCWH's analyses, the transport sector includes logistics, business travel and operational transport, which also includes the transport of patients and employees (9).

Some colleagues in different sectors of medicine, for example dermatology and urology, have shown that video consultations may reduce CO_2_ emissions. Studies in the medical specialty of orthopaedics and trauma surgery are still pending (10–12). The aim of the present study was to analyse the effect of video consultation on CO_2_ emissions using the specific situation during the covid-19 pandemic. We compared the estimated CO_2_ emissions in a 6-month period when conducting video consultations (VC) with that of a period with an exclusive face-to-face consultation in the outpatient clinic of the orthopaedics and traumatology surgery at a German university hospital.

## 2. Methods

### 2.1. Study participants

The study participants were patients who obtained a video consultation in the outpatient clinic of the department orthopaedics and traumatology surgery at a German university hospital in the period from June to December 2020 (Beginning of the Covid-19-pandemic in Germany) and voluntarily completed a questionnaire after the consultation. In the case of underage or mentally impaired patients (*n* = 2), the questionnaire as well as the supervision of the examination was carried out by the legally authorised person. The study comprised 51 consultations. The gender distribution was balanced in the patient collective (M:22; F:29, D: 1). The median age of the respondents was 51 years (2.7–87.9 years). In all cases of patients under 18 years of age, the consultation was conducted in the presence of the legal guardian, which also respond to the questionnaires. The video consultation are divided into spinal surgery, joint surgery, paediatric orthopaedics and accident surgery.

### 2.2. Study design and recruitment

All patients were sent an online questionnaire after the appointment. This questionnaire was anonymous and voluntary. It was divided into the following sections: personal data (age group, gender, place of residence), means of transport in case of face-to-face consultation (type of transport, journey), reason for the consultation (complaint region, type of consultation) and patient satisfaction. The response rate to the questionnaires was 60.71%. Only the data of those who had completed the questionnaire were analysed.

### 2.3. Calculation of Co_2_-emission

**Figure 1:**
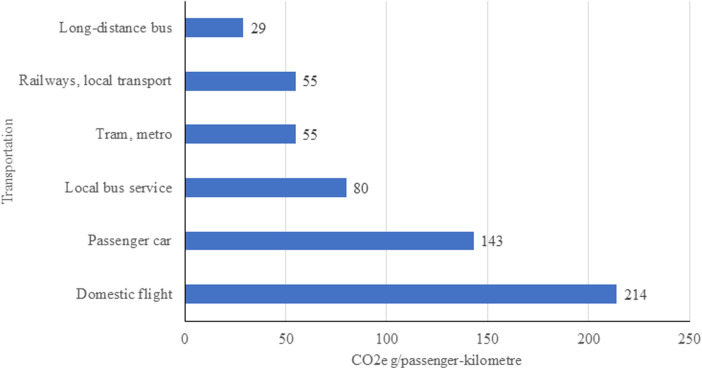
Co_2_e emission of transportation compiled after the German federal environment agency (6).

To calculate the greenhouse gas emissions, the route and the type of transport were considered. The calculation of the CO_2_ emissions was based on the data of the German Federal Environment Agency (4). These data sets include data on emissions of air pollutants from road traffic at regular intervals. This is published in the Handbook of Emission Factors (HBEFA). The German Federal Environment Agency has developed the computer programme TREMOD (Transport Emission Model) to calculate emissions for the period from 1960 to 2050. All passenger and freight transport modes are included in this calculation (13, 14). For our calculations, we used the estimates of the average emissions for transportation; assuming 143 g CO_2_e per person-kilometre for a journey by car (see Figure 1) (14).

**Figure 2:**
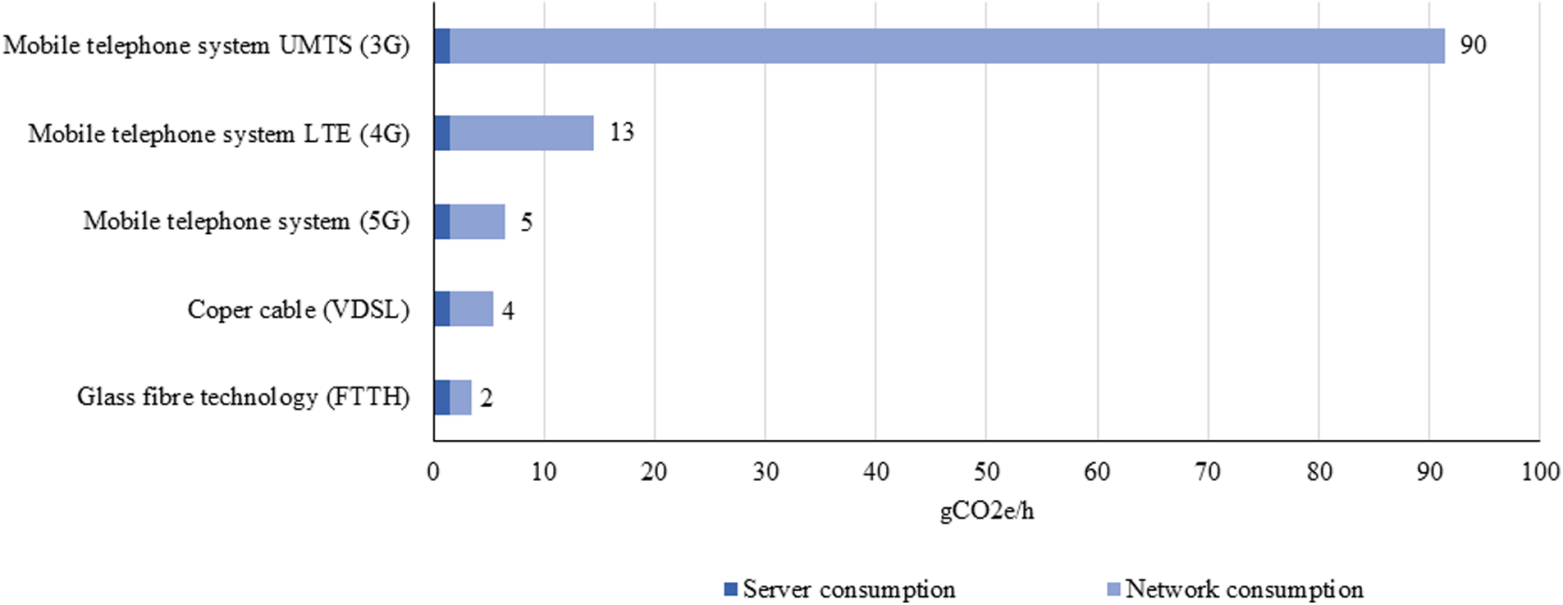
Co_2_e emission of data centre and network usage compiled after German federal environment agency (UBA) (15).

In order to adequately calculate the reduction of greenhouse gas emission, the emissions that occur during the performance of a video consultation were also considered (Figure 2). In particular, the power consumption of the data centres (server, storage systems, network and infrastructure) make up the majority of the emissions from video streaming (16, 17).

Fibre optic technology is environmentally most friendly with 2 g CO_2_e per hour of video streaming compared to the 3G network (UMTS “Universal Mobile Telecommunications System”) with 90 g CO_2_e (16, 17). In Germany, around 90 percent of households have access to “Very High Speed Digital Subscriber Line” (VDSL) connections with >50 Mbit/s (18). There are large differences between urban (>90%) and rural regions (43%) (19). Assuming that most German households have a VDSL connection, a 1-hour video consultation generates approx. 4 g CO_2_e per hour of video consultation (13, 18).

### 2.4. Outcomes

The primary endpoint of the study to evaluate the greenhouse gas emissions from a video consultation compared to the emissions from a face-to-face consultation. Secondary endpoints included satisfaction, time savings, and feasibility for different patients during a video consultation.

## 3. Results

**Figure 3:**
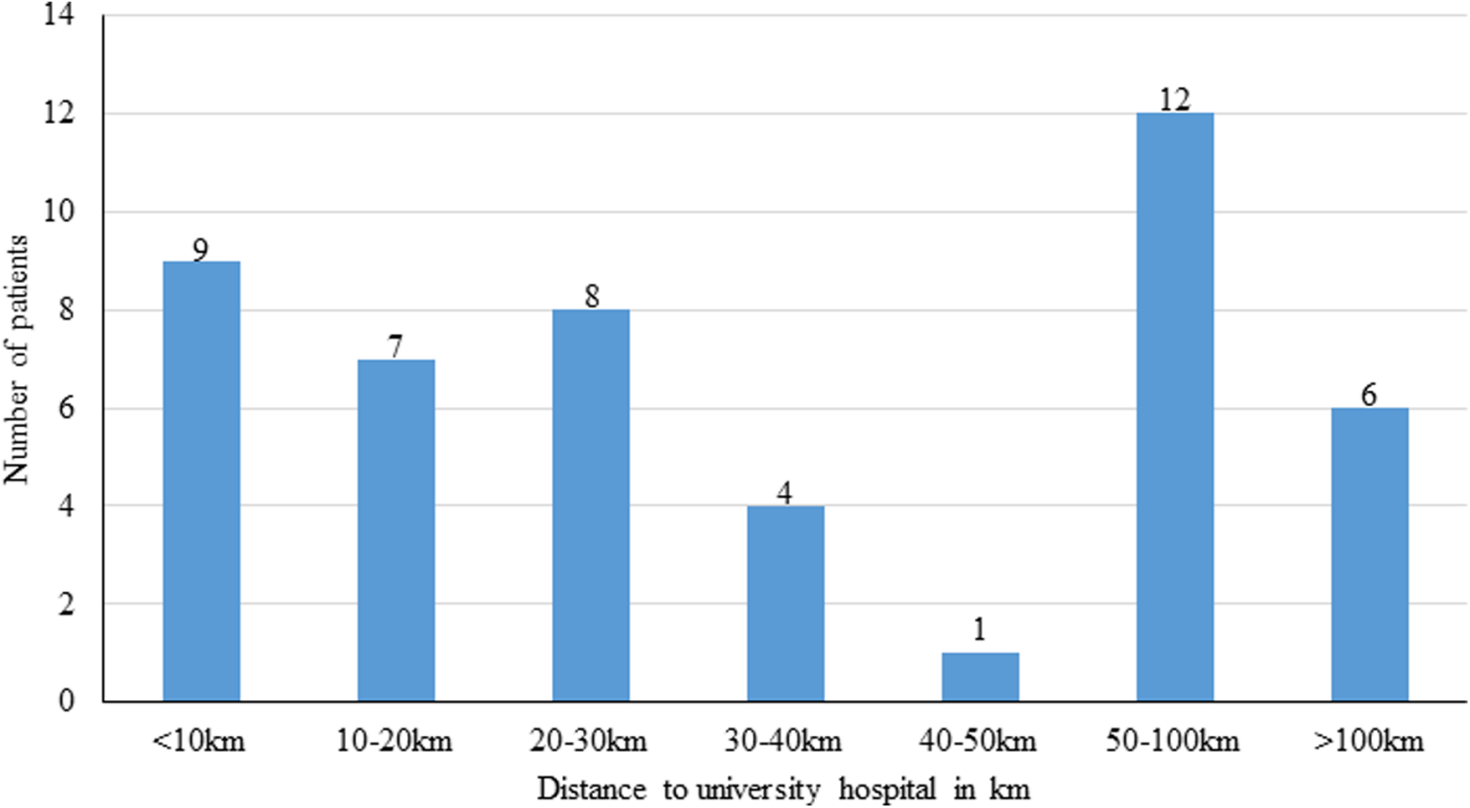
Travel distance of patients who took part in the study.

Among the 51 respondents 31% reported a travel distance less than 20 km to the clinic and 35% reported a travel distance of more than 50 km (Figure 3). The travel to the outpatient clinic would have taken less than 2 h for most patients (<1 h: *n* = 28; 1.5 h: *n* = 13; 2 h: *n* = 5; 2.5 h: *n* = 1; >3 h: *n* = 1). Most patients (74.5%) would have chosen to travel to the outpatient clinic by car. The search for a parking space would have taken about 30 min for most patients (64.7%). About 43.1% of the respondents were dependent on an accompanying person for a visit to our outpatient clinic. The most frequent reason (35.3%) for a consultation at the outpatient clinic was a follow-up. The majority of respondents (76%) conducted the video consultation at home and 9.8% at work. Many patients (43.1%) used their computer, 23.5% their tablet and 29.4% their smartphone for the consultation. On average, a video consultation lasted about 30 min. Two thirds of the respondents said that they would spend more than 2 h at every visit in an outpatient clinic for a face-to-face-appointment. A high percentage (84.3%) of the respondents were very satisfied or satisfied with the video consultation. Half of the respondents (*n* = 22) who completed a video consultation were dependent on an escort.

**Figure 4:**
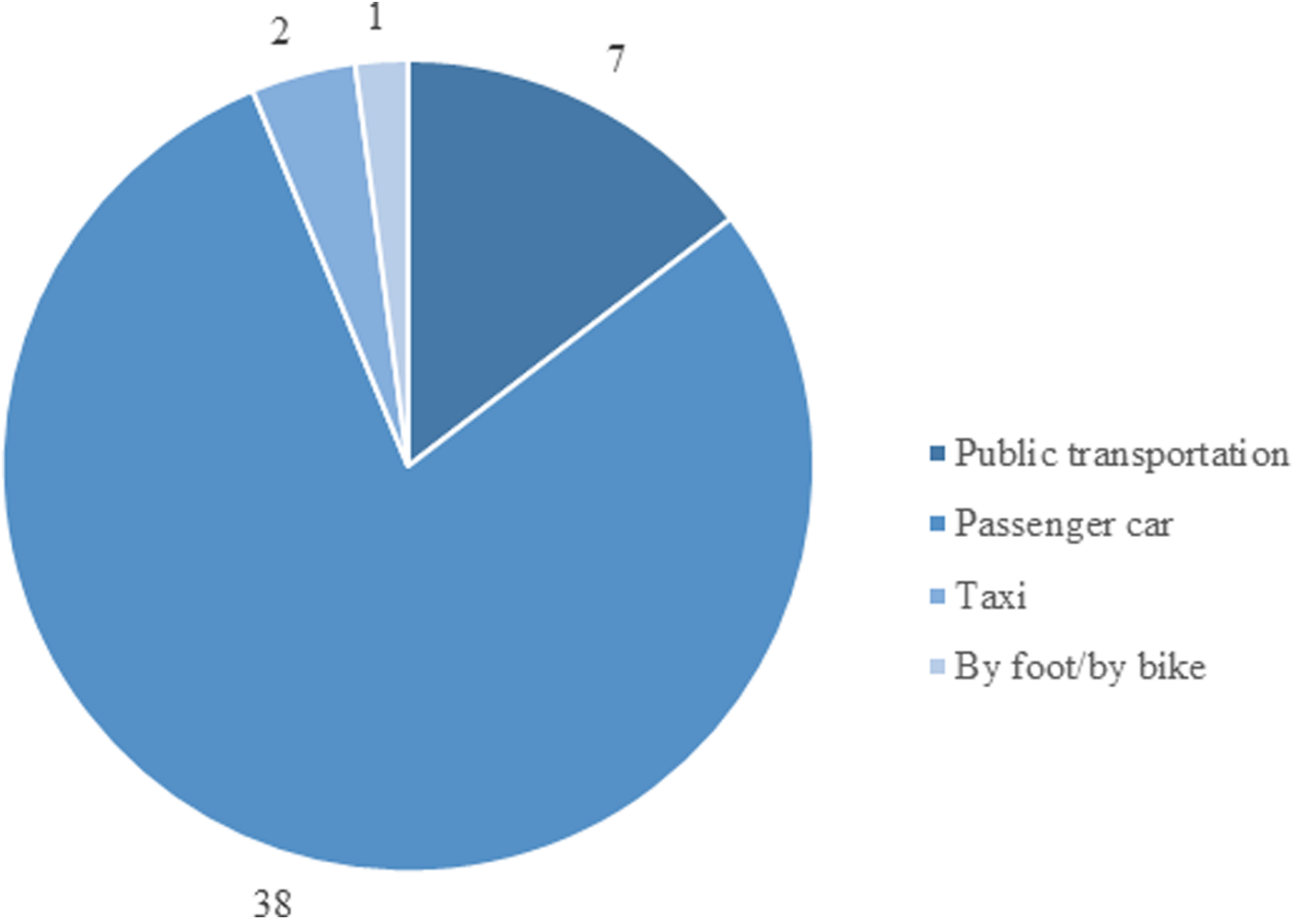
Choice of the mode of transportation by patients.

The data demonstrated a meaningful CO_2_ reduction, with a significant difference between the groups (*p* < 0.001). With reference to the data of the Federal Environment Agency, it was shown the implementation of the video consultation leads to a reduction in greenhouse gas emissions over 0.5 tons CO_2_e for the respondents compared to those patients traveling by car (Figure 4).

**Figure 5:**
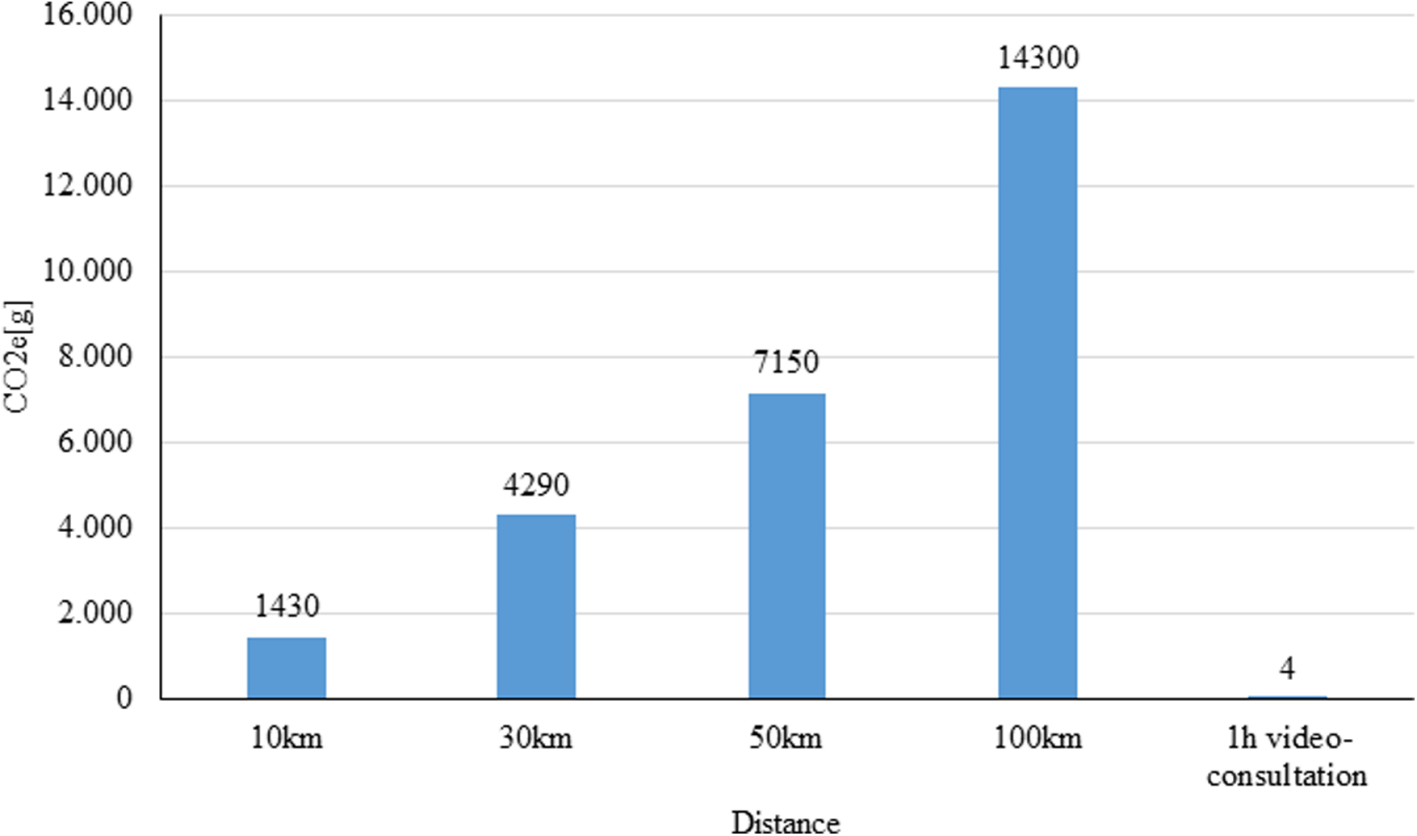
Calculated greenhouse gas emissions if patients would have travelled by car.

The most common reason for a video consultation was a follow-up for middle-aged patients with a journey to a special clinic in a distance of more than 30 km. A significant saving in emissions can be observed in patients who need to travel fort more than 10 km to the ambulance. The share of emissions by this group regarding the total emissions was 97.3%. Forty respondents would have driven to the outpatient department by car. On average, they would have to drive a distance of about 33 km (median 33.5 km; min 5 km, max 179 km). This would have resulted in around 0.5 tons CO_2_e. This is in contrast to 4 g CO_2_e used per one session of video consultation. The time the patient and the doctor were involved during the video consultation was included in the calculation. This resulted in a total of about 160 g of CO_2_e for all video consultations (Figure 5).

When 78.4% of the respondents would have travelled by car (incl. taxi), a total of 586,872 g CO_2_e would have be produced per return journey to the university's outpatient clinic. The high emission of patients who reported they would have use public transport to get to the clinic would have caused the emission of 15,299 g CO_2_e. Based on the current workload of the clinic's outpatient department with about 400 consultations per week, and assuming that about 20% of the consultations can also be performed online, it would significantly reduce greenhouse gas emissions.

## 4. Discussion

As one of the first estimates of the daily routine of the outpatient department for orthopaedics and trauma university hospital we have shown that, the implementation of video consultations can lead to a significant reduction in greenhouse gas emission. By running video consultations only, the German university clinic could reduce greenhouse gas emissions by 97% compared to face-to-face consultations. This result shows that there is a high potential for CO_2_ reduction in the implementation of video consultation.

The Federal Environment Agency of Germany estimated the total greenhouse gas emissions for Germany in 2019 to be about 810 GtCO_2_e. The emissions for the health care sector should have contributed about 42.12 GtCO_2_e (5.2% of national emissions) and the transport sector in the health care sector accounts for 2.95 GtCO_2_e (7% of emissions in the health care sector) (4, 6). Thus, reduction in this sector may significantly contribute to achieving the climate targets of Germany and the European Union. However, it is also important to mention that the use of digital media also has an impact on our environment through the power consumption of the servers and the use of rare earths for production. In addition, an electronic device have a limited lifespan and need to be replaced at regular intervals (16, 17). In order to make this technology even more environmentally friendly, demand-oriented planning should be aimed for. This includes the expansion of fibre optic cables, the sustainable operation of data centres and the economical use of mobile data transmission (16, 17).

Thanks to the video consultation, many patients can avoid potentially long journeys to a specialised university hospital for a second opinion or follow-up checks. A basic physical examination during a video consultation is possible or can be carried out by the general practitioner on site. Difficulties arise in particular with language barriers and pending imaging (e.g., sonography, x-ray diagnostics), where treatment may be delayed (20, 21). On the other hand, follow-up examinations in particular can be easily completed online, as the examiner is aware of the initial situation (22, 23). The video consultation also saves a lot of time for the patients and healthcare providers. On average, a video consultation lasts about 30 min. This is in contrast to the physical consultations, where the respondents reported a stay of about 2 h in our outpatient clinic with additional travel time (24, 25). The aim of the outpatient clinic is to avoid unnecessary in-person patient appointments and, if possible, to carry out a large part of the presentation by video consultation (26). The special challenge for orthopaedic patients is their limited mobility. Often these patients need an escort or help to get to the doctor. This is shown by the fact that half of the patients in our surveys were dependent on an escort. The video consultation can therefore also save the time for the escort.

In contrast to some other regions in Europe or Germany, the travel to a hospital in North Rhine-Westphalia (NRW) is within 8–12 min. for an ambulance and therefore comparatively short (27–29). In addition, there are many maximum-care hospitals in this region, so that a journey to a specialist is relatively short by European standards. For example, Buvik et al. describe distances of up to 250 km to a specialist in Norway (30). This is mainly due to the high population density and correspondent large number hospitals (344 in NRW as of 2017) (27, 30–32). The present study points out that the offer of a video consultation is a great advantage especially for patients who have a long journey (>50 km) to a specialised clinic. When talking about telecommunication, the still expandable access to the internet in the mainly rural regions should also be discussed. Patients in rural areas not only have to travel a long way to see specialists, but they also have limited opportunities for telemedicine. In Germany, less than 50% of the households in rural areas are connected to a data transmission rate of 50 Mbit/s or above (19). In order to increasingly establish telemedicine in Germany, this must be accompanied by an expansion of the digital infrastructure (13, 18, 33).

In their analysis of data from the National Health Service (NHS) UK, Wotton et al. divided the travel section into three groups: patient travel, visitor travel, staff travel and business travel. The total travel emissions were estimated to be about 49,000 tCO_2_e per year (34). This study showed, that over 40% of emissions due to travel were attributable to patient travel (34). Approximately one fifth was due to travel by healthcare workers. This shows the great potential for greenhouse gas reduction that exists in telehealth. Teleworking should also be expanded in the healthcare system. The SARS-CoV-2 pandemic has accelerated this development. The rapid changeover to video consultation would be possible because the telematic infrastructure had been intensified since a long time. On January 1st, 2016, the German Federal Ministry of Health stipulated the expansion of the digitalisation of medicine in the Social Code Book V in the “E-Health-Law”. In addition to the electronic doctor's letter and the electronic patient file, this also includes the offer of a video consultation. Telemedicine is one more step towards the digitalisation of medicine and thus towards more self-determined, individualised and modernised medicine. This obliges hospitals, health insurance companies and independent physicians to offer more telemedicine (20, 33, 35).

In addition to the reduction of greenhouse gas emissions from video consultation, the reduction of electricity consumption, waste production, and water pollution must also be considered to improve environmental protection (9–11). According to the HCWH report, the generation and distribution of electricity, gas and heat or cooling form the largest portion of greenhouse gas emissions in the healthcare system (9). In their analysis of emissions in the NHS, Conner et al. determined that 15% of emission is due to transport an another major part of emission (72%) by patient care is due to electricity consumption, waste production, and water pollution. Fathy et al. listed several possibilities for reduction of emissions such as the use of recycled paper, energy-inefficient office devices, or the use of water-saving systems, respectively (10). This shows where, apart from telemedicine, there is still a great potential for further consideration of environmental protection in medicine.

In various fields of medicine, such as urology and dermatology, studies have already been conducted on the influence of video consultation on emissions. Examples of this are the works of Croghan et al. and Fathy et al. (10, 35). Croghan et al. conducted more than 70% of their consultations in their clinic in the field of urology online in the context of the Corona pandemic. In their research, they showed that patients saved an average of 50 km of travel to the clinic, similar to our study. In accordance with our study, they showed that most savings occur when patients travel to the clinic by car (10, 35).

The present study refers exclusively to an outpatient clinic for orthopaedics and traumatology surgery. The feasibility must be adapted for different field of medicine (11, 22, 36). Due to the disease of the musculoskeletal system in orthopaedics and trauma surgery, recognition of instabilities, stiffening, degrees of movement etc. is essential. However, our study has shown that the video consultation can be used as a follow-up examination. The future aim should be to enable doctors and patients also to perform the initial examination as a video consultation. The video consultation should not be less qualitative and clinically informative than a face-to-face examination. The reason why in many cases colleagues still prefer a face-to-face presentation to a video consultation is the lack of standards and guidelines for the online examination of orthopaedic trauma surgery of patients. Currently, many working groups are trying to establish a standardised form for clinical diagnostics *via* video consultation (22, 23).

The video consultation offers the possibility of changing the daily routine at the clinic through the possibility of a work from home. In this way, the profession of doctor can be integrated into the modern, digital workplace. According to the research report of the German Federal Ministry of Labour and Social Affairs, mobile working leads to a significantly higher level of employee satisfaction (37). In addition, the doctors’ commute can be reduced, which can lead to a further reduction in CO_2_ emissions. This was not yet analysed in the study, as the doctors were in the hospital during the video consultation.

Since the protection of public health is the most important task of the health care system and CO_2_ emissions have such a great impact on public health, the reduction of emissions in terms of transportation, energy consumption, waste production and water pollution is of fundamental importance (9, 10, 38).

## 5. Limitations

One point of criticism of the study could be the imprecise data on the data transfer rate of the patients, but a significant expansion of the telecommunication infrastructure can be expected in the metropolitan areas. In rural regions, access to broadband connections is often not available (18). It should also be noted here that the age group between 40 and 65 is particularly well represented. The reason for this is likely the lack of digital competence among the elderly and geriatric patient group. However, this patient group represents the majority of the patients in most departments. In addition, it should be mentioned in the projections that the transport sector in the health care system not only includes the travel of patients, but also other types of transport, such as the travel of staff, visitors, etc (2). Video consultation cannot be carried out for all consultations because in some cases a personal examination is indispensable. A disadvantage of the digital consultation is the lack of possibilities for physical examinations in the case of musculoskeletal diseases. In addition, direct diagnostic imaging is not possible, which can lead to a delay in the correct treatment. Also, in the case of language barriers, video consulting can only lead to limited results.

## 6. Conclusion

In our study, we have shown that conducting a video consultation leads to a 97% reduction in greenhouse gas emissions compared to a face-to-face presentation in our model investigation of an outpatient clinic of orthopaedics and traumatology surgery. Video consultations are an important step towards more environmental protection in medicine by significantly reducing greenhouse gas emissions. The video consultation can be an important part of the reduction of greenhouse gas emissions in the health care system, it also saves time for the doctor and patient and is an important part of individual patient care.

So far, there are no guidelines and standards for the digital examination of orthopaedic and trauma surgery patients in Germany; these will have to be worked out in the near future.

## Data Availability

The raw data supporting the conclusions of this article will be made available by the authors, without undue reservation.
